# Effects of exposure to 2100 MHz GSM-like radiofrequency electromagnetic field on auditory system of rats^☆^

**DOI:** 10.1016/j.bjorl.2016.12.002

**Published:** 2017-01-27

**Authors:** Seyed Mohammad Javad Mortazavi, Seyed Ali Reza Mortazavi, Maryam Paknahad

**Affiliations:** a Shiraz University of Medical Sciences, School of Medicine, Medical Physics Department, Shiraz, Iran; b Shiraz University of Medical Sciences, Ionizing and Non-ionizing Radiation Protection Research Center (INIRPRC), Shiraz, Iran; c Shiraz University of Medical Sciences, School of Medicine, Research Committee, Shiraz, Iran; d Shiraz University of Medical Sciences, School of Dentistry, Oral and Maxillofacial Radiology Department, Shiraz, Iran

Dear Editor,

We have read the article by Celiker et al. entitled “Effects of exposure to 2100 MHz GSM-like radiofrequency electromagnetic field on auditory system of rats” that is published in the Brazilian Journal of Otorhinolaryngology.^1^ Celiker et al. evaluated the effects of 2100 MHz Global System for Mobile communication Electromagnetic Field (EMF), generated by an EMF generator on the auditory system of rats. Despite its challenging theme and well-structured content, the paper authored by Celiker et al. has some shortcomings. The first shortcoming of this study comes from this point that the authors have only investigated the effects of 2 modes (i.e. talk mode and switched-off mode). Therefore, data about 2 other remaining modes of “standby” and “Talk + Wi-Fi” (talking using a mobile phone that is connected to the Wi-Fi network) are missing. It is worth noting that in contrast with the earlier reports,^2^ it has been shown that personal exposure to EMF can be affected by one's own mobile phone in stand-by mode.^3^ Furthermore, due to rapid advances in telecommunication technology, mobile phones in modern life are much more frequently used for surfing the Internet than calling. Therefore, we usually use our mobile phones in the talk-mode while it is connected to the Wi-Fi network, simultaneously.

Another shortcoming of this study is also due to the technical properties of the EMF generator used in this study. The authors stated that “The RF-EMF group was exposed to a continuous EMF produced by an EMF generator (Anritsu MG3670B, Japan) for 30 days. The generator was adjusted at a signal level (power) of 5.4 dBm (3.47 mW) and a frequency of 2100 MHz to simulate the talk mode on a mobile phone.” It should be noted that the MG3670B digital modulation signal generator which operates in the frequency range of 300 kHz–2.25 GHz is fundamentally designed for testing and evaluating the digital mobile communications equipment and related devices. In this light, the authors should provide some basic information about modulation (in case this device is used with modulation) and other technical specifications which show that MG3670B could simulate a GSM mobile phone.

## Conflicts of interest

The authors declare no conflicts of interest.
